# Versatile knowledge guided network inference method for prioritizing key regulatory factors in multi-omics data

**DOI:** 10.1038/s41598-021-85544-4

**Published:** 2021-03-24

**Authors:** Christoph Ogris, Yue Hu, Janine Arloth, Nikola S. Müller

**Affiliations:** 1 Institute of Computational Biology, Helmholtz Center Munich, Ingolstädter Landstr. 1, 85764 Neuherberg, Germany; 2 Department of Translational Psychiatry, Max Planck Institute of Psychiatry, 80804 Munich, Germany

**Keywords:** Computational biology and bioinformatics, Systems biology, Biomarkers

## Abstract

Constantly decreasing costs of high-throughput profiling on many molecular levels generate vast amounts of multi-omics data. Studying one biomedical question on two or more omic levels provides deeper insights into underlying molecular processes or disease pathophysiology. For the majority of multi-omics data projects, the data analysis is performed level-wise, followed by a combined interpretation of results. Hence the full potential of integrated data analysis is not leveraged yet, presumably due to the complexity of the data and the lacking toolsets. We propose a versatile approach, to perform a multi-level fully integrated analysis: The Knowledge guIded Multi-Omics Network inference approach, KiMONo (https://github.com/cellmapslab/kimono). KiMONo performs network inference by using statistical models for combining omics measurements coupled to a powerful knowledge-guided strategy exploiting prior information from existing biological sources. Within the resulting multimodal network, nodes represent features of all input types e.g. variants and genes while edges refer to knowledge-supported and statistically derived associations. In a comprehensive evaluation, we show that our method is robust to noise and exemplify the general applicability to the full spectrum of multi-omics data, demonstrating that KiMONo is a powerful approach towards leveraging the full potential of data sets for detecting biomarker candidates.

## Introduction

Over the past decade, inventions of high throughput techniques enabled the measurement of multiple omic levels on a large scale and in a cost-efficient manner. The resulting data provides deep insights into the network of molecules orchestrating biological mechanisms. Hence, it is no surprise that many studies underpin their findings using multi-omic analysis when identifying disease or condition specific key molecules. So far, there exist several different multi-omic analysis strategies. Probably one of the first and well-established combinations of two omic levels, are expression quantitative trait loci (eQTL) analyses^1^. However, integrating multiple levels simultaneously is still an ongoing challenge. To account for this, most studies use a level-by-level approach where each omic level is analysed independently. Disregarding the complex ‘cross-omic’ interplay simplifies the analysis problem but comes at the cost of potential overlooked and misinterpreted results^2,3^.

Recently, more sophisticated latent factor-based models have been introduced, capable of analysing multiple omic levels simultaneously^4,5^. These methods infer lower-dimensional representations (latent factors) of the original high dimensional multi-omic data space. Even though the latent factors represent certain patterns of the data, it is often difficult to find the biological meaning of these factors^5^. Improved interpretability is one of the big advantages of network based approaches. These identify condition specific key molecules via inferring and analysing a network representation of the processes^6,7^. Such networks often consist of thousands of nodes, representing the different omic input features, such as genes and proteins which are linked condition-specifically.

The most common and straightforward inference approaches generate correlation based networks. Here network links are generated by calculating pairwise correlation between all features. Unfortunately, this strategy often results in low-specific and highly connected networks which are hard to interpret^8^. To increase the specificity one can use more advanced machine learning approaches, instead of correlation, to identify associations between nodes^9,10^. These methods are only applicable to high dimensional multi-omic data with large amounts of samples.

To overcome the limitation of relying on pairwise correlation we previously developed miRlastic^11^. MiRlastic facilitates prior knowledge to increase the performance for high dimensional and low sample size data analysis. MiRlastic creates a multivariate penalized regression model for each mRNA with the multitude of predicted miRNA regulators per mRNA, thereby filtering predicted regulations in the context of real data. Next, aggregating miRNA and mRNA species from all regression models assembling a mRNA-miRNA interaction network.

We now developed a method capable of using any kind of omic levels inferring a multi-level network. Our novel Knowledge guIded Multi-Omic Network inference method—KiMONo, vastly expands miRlastic. KiMONo allows a simultaneous integration of multiple omics levels, for example, genomic variants, methylation, gene expression, proteomics and biological information. KiMONo leverages various prior information, reduces the high dimensional input space, and uses sparse group LASSO (SGL)^12^ penalization in the multivariate regression approach to model each gene's expression level. The chosen penalty term allows for a bi-level selection, penalizing each omic level as well as penalizing within each level. Aggregating these models, all features are linked to their gene and assembled in the final heterogeneous multi-level network.

In our benchmark were able to showcase KiMONo’s performance by applying it to one of the most comprehensive multi-omic data sets available—the PanCancer data collection of The Cancer Genome Atlas. It consists of almost 5000 samples describing 12 different cancer types across ~ 50,000 features on five omic levels. As a validation we also applied the same benchmark setup to a data set of the highly complex disease major depressive disorder (MDD). This challenging data set contained over 107 samples measured on four different omic levels with in sum over 4.5 million features. Finally, we successfully identified key molecules driving the conditions within these data sets. KiMONo retrieved previously reported genes that matched the underlying disease setting, in addition to yet unknown potentially interesting genes. We present KiMONo as a versatile method to derive fully integrated and holistic multi-level networks capturing the data-supported interplay between omics levels.

## Results

### Network inference with KiMONo

**Figure 1:**
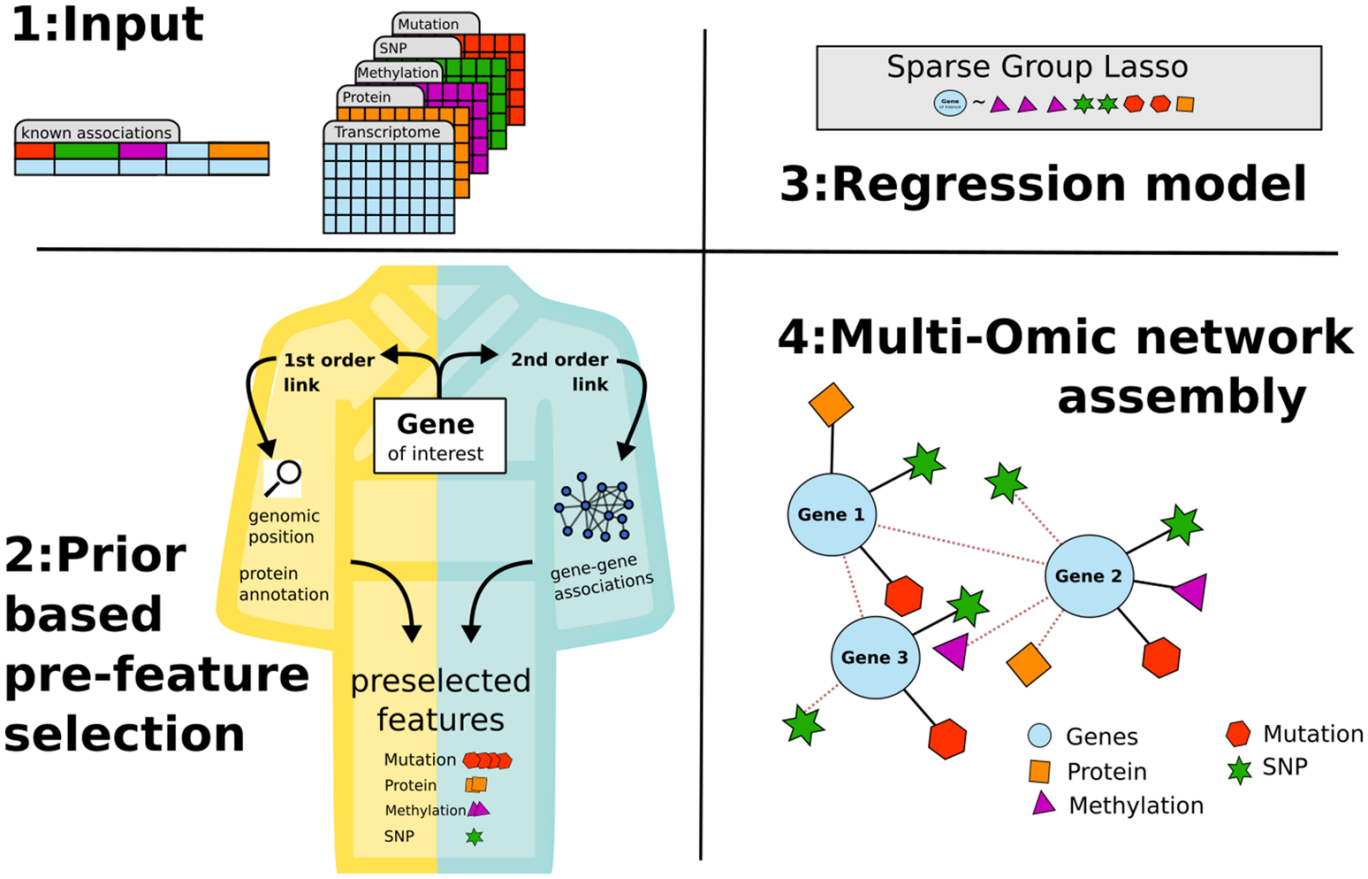
Workflow. 1:Input—the input data for KiMONo can be any mix of multiple omic data and prior knowledge. The prior represents general biological knowledge and is submitted via a list of already known associations between input features. 2:Prior based pre-feature selection—Based on the prior, KiMONo pre-selects omic features and generates a input matrix *X* for each gene. 3:Regression model—Each gene is modeled via a sparse group lasso using the genes expression as *y* and the previously selected matrix *X* as input. 4:Multi-Omic Network—all gene models are merged to generate a multi-level network containing features from all input sources as nodes and links for all non-negative regression coefficients between them.

Our novel method KiMONo infers a condition specific multi-level network from any mix of multi-omic data sets (see Fig. 1). The network nodes represent omic input features, like genes or proteins, linked if a regulatory effect is present within the data. KiMONo’s efficient inference is achieved by using existing biological knowledge to pre-select features of different omic data types for a gene of interest. The condition-specific information is inherent to the data used for inference. This biological knowledge, or prior, can range from an experimentally validated interaction between proteins up to simple annotations between genes coding for proteins. This can be interpreted as a blueprint, used to further focus and guide the algorithm. For KiMONo the prior has to be submitted in a list format including all already known, thus biologically possible, associations. Within the prior knowledge, KiMONo can also differentiate between direct (first-order) and indirect (second-order) associations. For instance, first-order links can describe the relation between a protein complex and one of its coding genes and relations between all coding genes can be implemented as second-order links.

Once the prior and input data are submitted, KiMONo optimizes a penalized regression model for nodes individually to establish its outgoing network links. The feature’s value, i.e. gene expression, represents the criterion variable $y$ while the input matrix $X$ is assembled by the features associated to the gene within the prior. KiMONo uses the SGL regression approach to penalize within and between predefined groups of features. By performing this ‘bi-level’ selection, KiMONo accounts for different underlying distributions between the features originating from using multiple data types. Within SGL, the parameters $\alpha$ denotes the intergroup penalization while $\tau$ defines the group-wise penalization. KiMONo approximates an optimal parameter setting via using the Frobenius norm^11^. To be more specific,$\alpha$ is approximated by the mean Frobenius norm of all groups while $\tau$ is estimated by the frobenius norm within each omic level. The global LASSO parameter $\lambda$ is optimized using a fivefold cross-validation, using the mean squared error as loss function.

KiMONo further uses the fitted models, of all nodes to assemble a multi-level omic network. Within this network, nodes represent features of the input data, like genes, proteins methylation sites or SNPs, and connections between them are weighted via the $\beta$ coefficient. Furthermore we assign each model a confidence score based on its evaluated ${R}^{2}$.

### Increased performance using second-order links

We used the breast invasive carcinoma data, a subset of PanCancer collection, as a benchmark set. It is the largest set containing 604 patients with ~ 50,000 measured features across the 5 omic levels described in the previous section.

First, we evaluated the performance using all features at once without any prior pre-selection. Since, neither conventional LASSO or elastic net nor SGL were able to infer networks we asked next whether incorporating first- and second-order links would increase KiMONos inference performance?

An evaluation of the inferred networks using only the first-order prior showed that 5,349 models were inferred beyond intercept-only modes having a mean ${R}^{2}=0.02$. Only 96 gene models performed with ${R}^{2}\ge 0.02$. But, once we expanded the models with second-order associated features, the performance considerably increased: 9480 gene models with successful feature selection showed an ${\overline{R}}^{2}=0.11$ of which 3,150 models performed better than ${R}^{2}\ge 0.1$ with an ${R}^{2}=0.25$.

**Figure 2:**
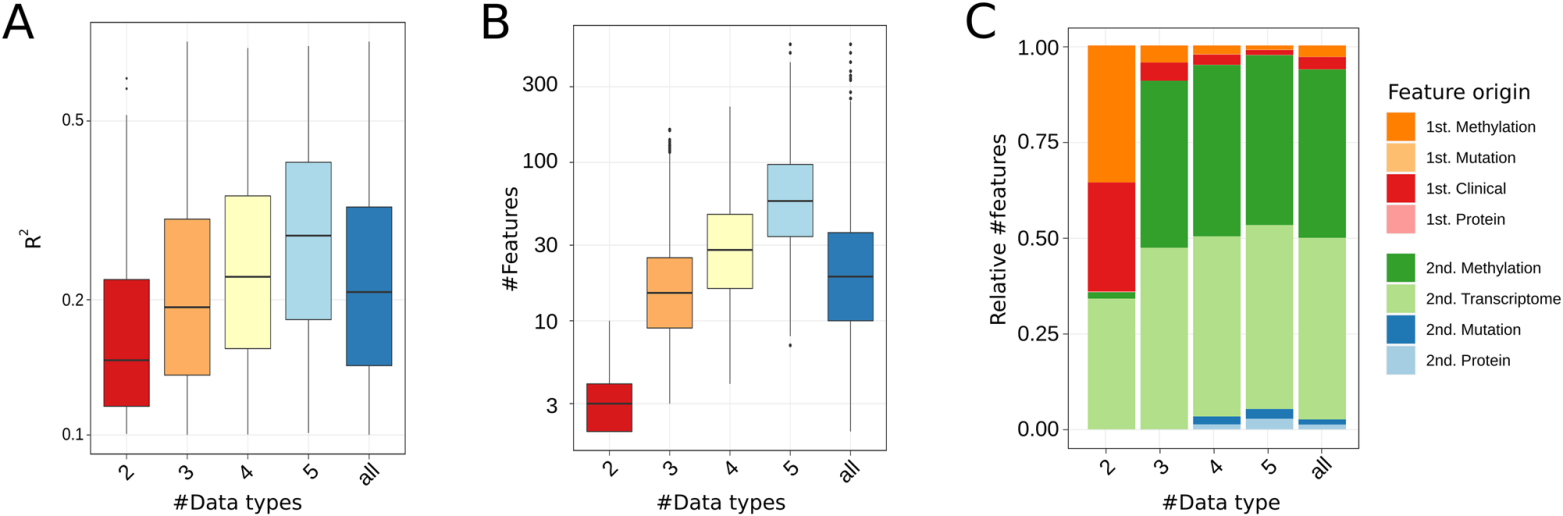
(**A**) Boxplots of performances of models composed of features from 2, 3, 4, and 5 different data types/omic levels, as well as the overall performance over all levels. (**B**) Number of features selected when selecting features of different data types, as well as the general number of features used over all levels. (**C**) Composition of omic types selected in models. Here orange-red refers to 1st order linked features while green and blue visualize 2nd order features.

Next we evaluated if the amount of different omic layers also impacts the performance. Therefore, we selected models with second-order prior and ${R}^{2}\ge 0.1$ and grouped models according to the number of different data types retained in the selected features (Fig. 2A). No model was composed of a single data type. Models based on features originating from two omic levels showed an average ${\overline{R}}^{2}=0.19$ while five omic layers increased the ${\overline{R}}^{2}=0.3$. Furthermore we can also observe that the increased performance with increasing number of data types is also related to an increased number of features used, see Fig. 2B. Here the majority of models, which used composed of features from two omic levels with average number of 3.6 linked features, while models using data of five different omic sources detected an average of 77.5 selected features. Moreover we can observe that 2nd order selected features are dominating the final models. Here transcriptomic and methylation based features are dominating the generated models, see Fig. 2C.

### Performance on small sample-sized data

**Figure 3:**
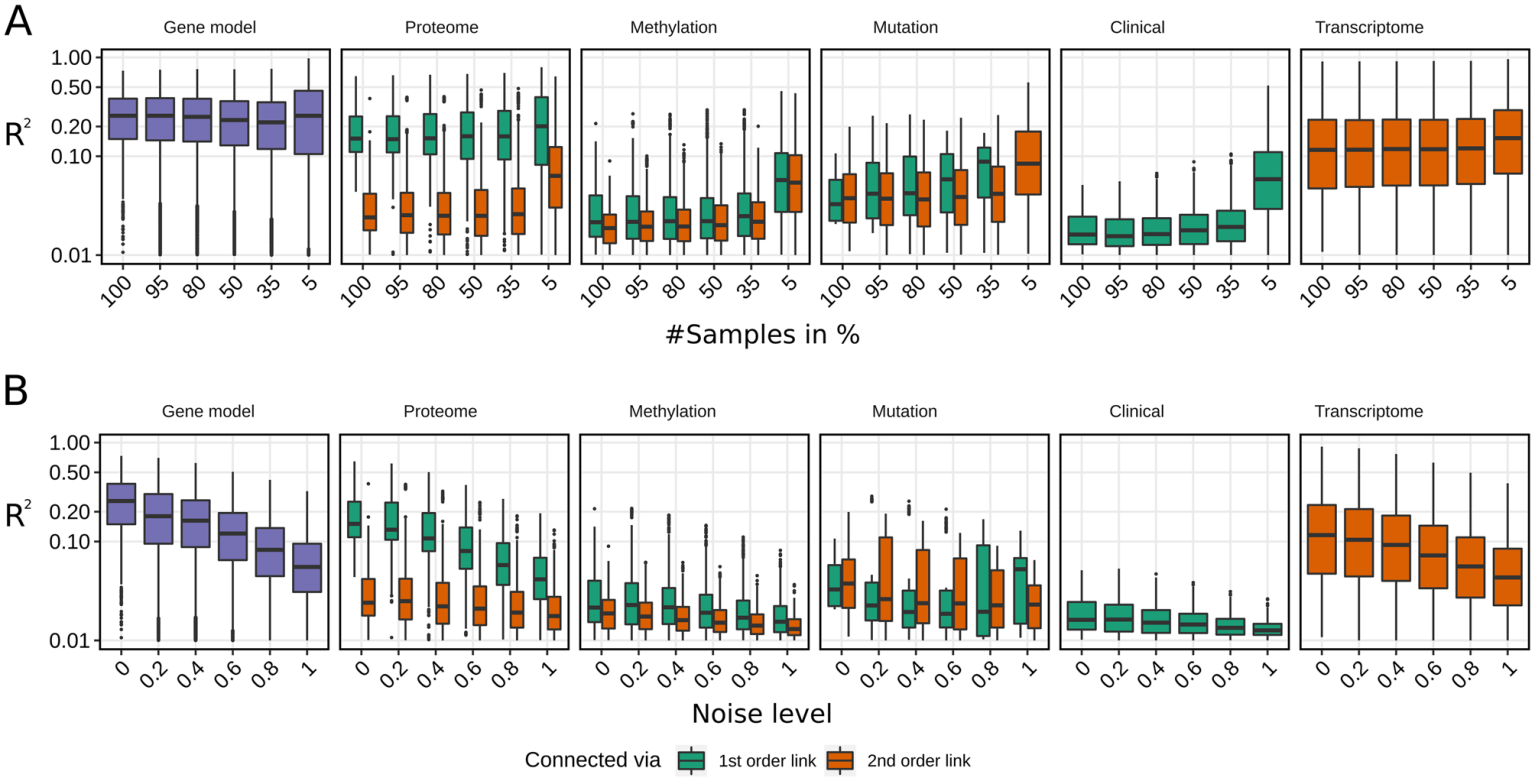
Robustness benchmark for (**A**) different sample sizes and (**B**) noise levels. First set of box plots (purple) shows the overall performance,${R}^{2}$(log scaled y axis) of inferred gene models using all available information layers. All following sets describe the stand-alone performances of Proteome, Methylation, Mutation, Clinical and Transcriptomic information layers. 1st order links (green) and 2nd order links (orange) are benchmarked separately. Note, Clinical and Transcriptome information consists of only 1st and 2nd order links. (**A**) Data sets with different sample sizes were generated using 5–100% of the 604 breast invasive carcinoma samples. (**B**) Different test data sets were simulated by adding Gaussian noise with increasing variance to the scaled feature values. Here, the noise level reflects the ${R}^{2}$ for six intensities.

One of the biggest challenges for multi-omic data analysis methods is preserving robust performance on multi-omic data sets with low sample size. To benchmark if KiMONo is affected by low sample size data, we simulated in total 100 test sets based on *PanCancers’s breast invasive carcinoma* data. Using KiMONo we inferred a network for each test case where 5–95% of the samples were removed (Fig. 3A), and compared them to a reference network inferred on all samples. Interestingly, 80% of the initial higher-performing models (${R}^{2}>0.1$) were inferrable even when we only use 5%, 30 of the 604, samples (Fig. S3A).

To evaluate the overall performance we excluded genes models which explained less than 1% of the gene expression variance (${R}^{2}<0.01$) and restricted the benchmark set further to 932 genes which have been also present in the 5% test case (an unrestricted view can be found in Fig. S3). Removing samples also decreased the variance of many features which indirectly decreased the overall dimensionality. Using 30 (5%), of the 604 samples, reduced the number of features from 57,966 to 15,632 features. Comparing the overall results we showed that KiMONos performance was stable for both large and small data sets. The reduction of variance and thus complexity was reflected by a slight increase in performance between the 35% and 5% test cases, from ${R}^{2}=0.24$ to 0.29.

Following our approach of dissecting the overall ${R}^{2}$(see Materials and Methods) we were able to estimate the importance of the individual levels as well. The most informative sole information layer was the *Protein* information with and average ${R}^{2}\hspace{0.17em}$= 0.23 followed by the second-order linked *Transcriptome* information with an average ${R}^{2}$= 0.18. The sparse *Mutation* data seemed to improve its performance with smaller sample sizes whereas *Clinical*, *Methylation* and second-order linked *Protein* information seemed to contribute the least. When comparing the results between the different sample sizes, the mutation layer constantly improved the performance by ${R}^{2}$ of 0.7 for reduced sample sizes with lower dimensionality while all other information layers slightly decreased in performance.

### Performance on noisy data sets

Another major challenge for data analysis methods is coping with noisy data sets. To evaluate this we simulated noisy variations of the *breast invasive carcinoma* data. Following the simulation approach described in Materials and Methods, we generated 100 different data sets across five noise levels. Using KiMONo we inferred networks for all test cases and again compared it to a reference network inferred of the *breast invasive carcinoma* data without noise.

We observed a strong effect of noise on the coverage of the network (Fig. S2B). Looking at the highest noise level of $\alpha =1$ KiMONo was able to still retrieve more than half (4071) of the initial models with ${R}^{2}>0.01$. Looking at higher-performing models (${R}^{2}>0.1$), the gene coverage dropped from 3147 models to 463. This drop in coverage was also observed by evaluating the overall gene model performance. Here, we only evaluated models explaining at least 1% of the variance within the gene expression. The most drastic impact of noise can be observed at the 1st order linked *Proteome* and 2nd order *Transcriptome* data (Fig. 3B). In the *Proteome* data, the performance dropped from 0.21 to 0.05 while the *Transcriptome* decreased from 0.17 to 0.6. The overall average ${R}^{2}=0.28$ was decreased to 0.05, after adding Gaussian noise with $\alpha =1$. Similar to the previous performance test, there was a similar overall trend over all other information levels. Information levels that already started with a relatively low ${R}^{2}$ like *Methylation (0.03)* and *Clinical* (0.02) layer, maintained the general low performance of 0.02 and 0.01, respectively.

### Multi-layer PanCancer networks

**Figure 4:**
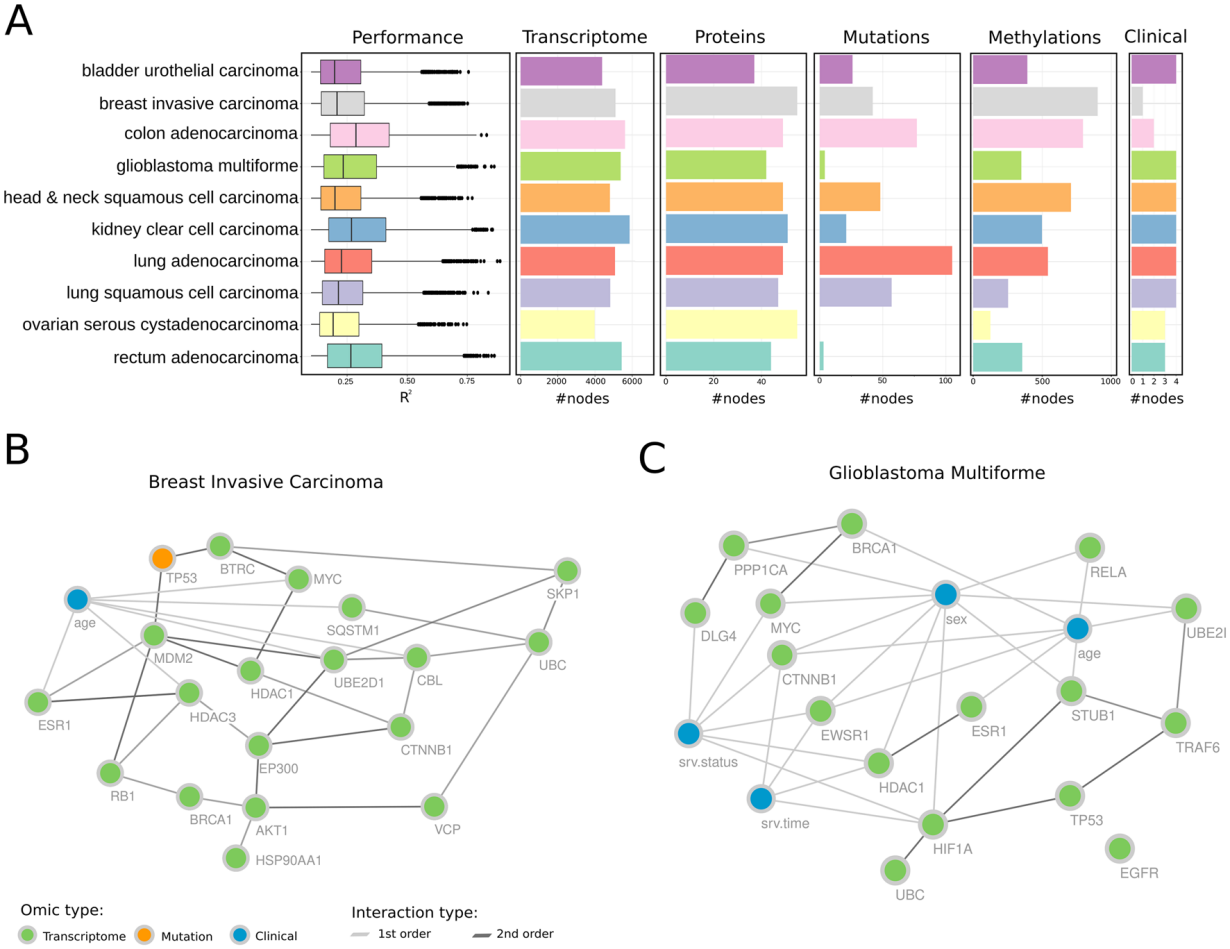
(**A**) Performance on all gene models (${R}^{2}>0.1$) inferred by KiMONo followed by number of gene models and number of features selected in the proteomics, mutation, epigenetic and clinical data layer. (**B**) Subnetwork of top 20 features (highest betweenness of centrality) within the inferred breast invasive carcinoma network (${R}^{2}=0.4$). Within the network, we found nodes originating from mRNA (green), mutation (orange) and clinical (blue) feature space. The edges denote first order edges (grey), first and second-order (black). (**C**) Subnetwork of the top 20 features in Glioblastoma Multiforme (${R}^{2}=0.4$).

To exemplify the data analysis power of KiMONo on multi-layer data, we inferred networks on the PanCancer data consisting of 11 cancer types. As a post-processing step, we excluded all models for which ${R}^{2}<0.1$ and also excluded links within the network with a weight smaller than $\beta <0.02$, Fig. 4A.

The final networks had on average 26.343 links and 3158.2 nodes (Fig. 4A). The test for degree distribution yielded a significant gamma distribution ($p<2.2e-16$). For each network, we ranked the nodes based on the node betweenness of centrality and selected the top 100. Comparing these sets showed that 88% of the top 100 nodes are occurring in at least two of the cancer types. All genes which were identified as important across all 11 cancer networks had been previously linked to cancer by several studies (see Table S1). We further used all those genes for pathway annotation using the open source tool pathwaX^13^. Here the top enriched KEGG pathway is the cancer-related *Chronic myeloid leukemia* (FDR = 1.45e−37) pathway followed by *Pathways in cancer* (FDR = 6.3e−35). Furthermore, we were able to identify 345 features which were uniquely identified to each cancer type. For instance, the methylation site cg00103783 (chr17:7.583.931), mapping to *MPDU1* gene, was only detected as important within the *head & neck squamous cell carcinoma* network. Interestingly^14^, introduced MPDU1 as a potential biomarker for HNSC.

Within the breast invasive carcinoma network, all three genes were among the top 20 nodes, lead by *age* and *UBC* which had been identified as an oncogene by^15^ (see Fig. 4B)*.* Using these top 20 genes for pathway annotation gave a clear picture of cancer-related KEGG pathways, i.e.: KEGG *Pathways in Cancer* (FDR = 2.94e−44) was the top enriched pathway, followed by *Hepatitis B* (FDR = 2.51e−39) and *Cell cycle* (FDR = 2.3e−38). Both, *Cell cycle* and *Hepatitis B,* were known breast cancer-related pathways^16,17^. However, the *Breast Cancer-specific* KEGG pathway ranked on place 14 (FDR = 5.0395e−33) among all enriched pathways. Another interesting result was the inferred *Glioblastoma multiforme* (GBM) network, Fig. 4C. Even though *GBM* is one of the rarest cancer types, it is also one of the most lethal ones having a survival time of 14–15 months after diagnosis^18^. The *GBM* data set was relatively small including only paired data for 61 patients with 58,051 features across 5 omic layers. Nevertheless, KiMONo inferred 112,945 links between 9341 nodes. Even though the top 20 features were not as densely connected as in the previous example, we were able to link *CTNNB1, HIF1A*, *HDAC1 and EWSR1* to *survival time.* Beside *EWSR1* all have been reported as survival time related in GBM (^19,20^. Interestingly^21^ showed that in GBM, EWSR1 was often fused with *PATZ1, a cancer related gene,* worsening the survival rates^19,22^.

### Multi-layer MDD network

Even though the PanCancer is one of the most comprehensive multi-omic datasets available, we further wanted to evaluate our method on a more complex type of disease, like MDD. While progress has been made in understanding the pathomechanisms of MDD, success in translating findings into clinical practice has been limited^23^. Studies have been largely focused on single-level omics, like GWAS^24^) and multi-level omics are relatively new^25,26^. Therefore, making successful inference of a multi-omic cross-talk regulatory network is of importance to better understand the depression phenotype.

For this purpose, we applied KiMONo on a cohort, consisting of 107 healthy individuals and patients. There were 4,247,909 imputed SNPs, 12,418 transcripts and 320,481 methylation sites available for the evaluation of our method, after filtering for the 25% of methylation sites with the least variance. Biological information such as BMI, age, sex and status of the diagnosis and cell type composition were also taken into account for network inference.

**Figure 5:**
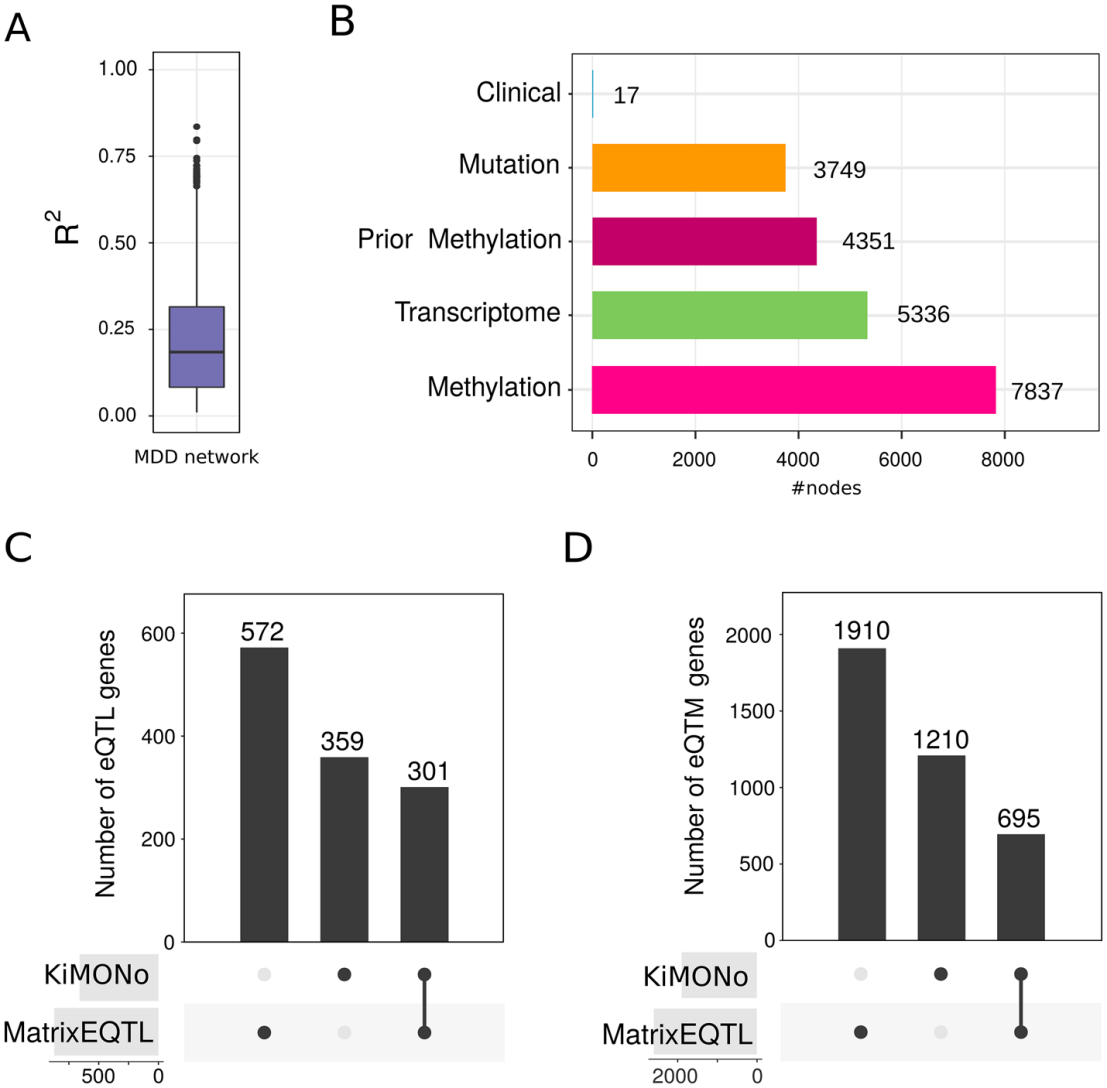
(**A**) MDD network Performance on all gene models n = 9943 inferred by KiMONo after filtering for − 0.02 < $\beta$< 0.02 coefficient and ${R}^{2}>0.1$. (**B**) Composition of retained features deriving from omic levels of first-methylation and SNPs, as well as and second-order methylation, SNPs, gene expression and biological clinical features; comparison of (**C**) number of eQTL genes and (**D**) eQTMs gene derived from KiMONo and matrixEQTL.

To ensure a higher quality of selected features we filtered for $\beta$ coefficients between − 0.02 and 0.02 as well as ${R}^{2}<0.1$ values. The final MDD network, comprised of 9,943 gene models with median ${R}^{2}=0.184$ of which few models even reached very high ${R}^{2}>0.75$ values,. As predictors, we uncovered 7837 methylation sites and 3749 SNPs as first-order links, as well as 5336 gene transcripts and 4351 methylation sites as second-order links. In addition, all of the biological covariates were found across the whole network (Fig. 5A,B).

To compare with state-of-the-art methods, we identified eQTL and eQTM genes using pairwise models and set them into context to the findings of KiMONo. Using the same proximity restrictions for the MatrixEQTL and KiMONo, we found 873 and 660 eQTL-genes, respectively, overlapping in 301 (Fig. 5C). Further, we found an overlap of 695 eQTM genes, with 1210, more than double found with KiMONo (Fig. 5D). Nearly all genes found in the overlap or only by KiMONo were further explained in multivariate models by information from other omic-layers of methylations, SNPs and gene expression.

**Figure 6:**
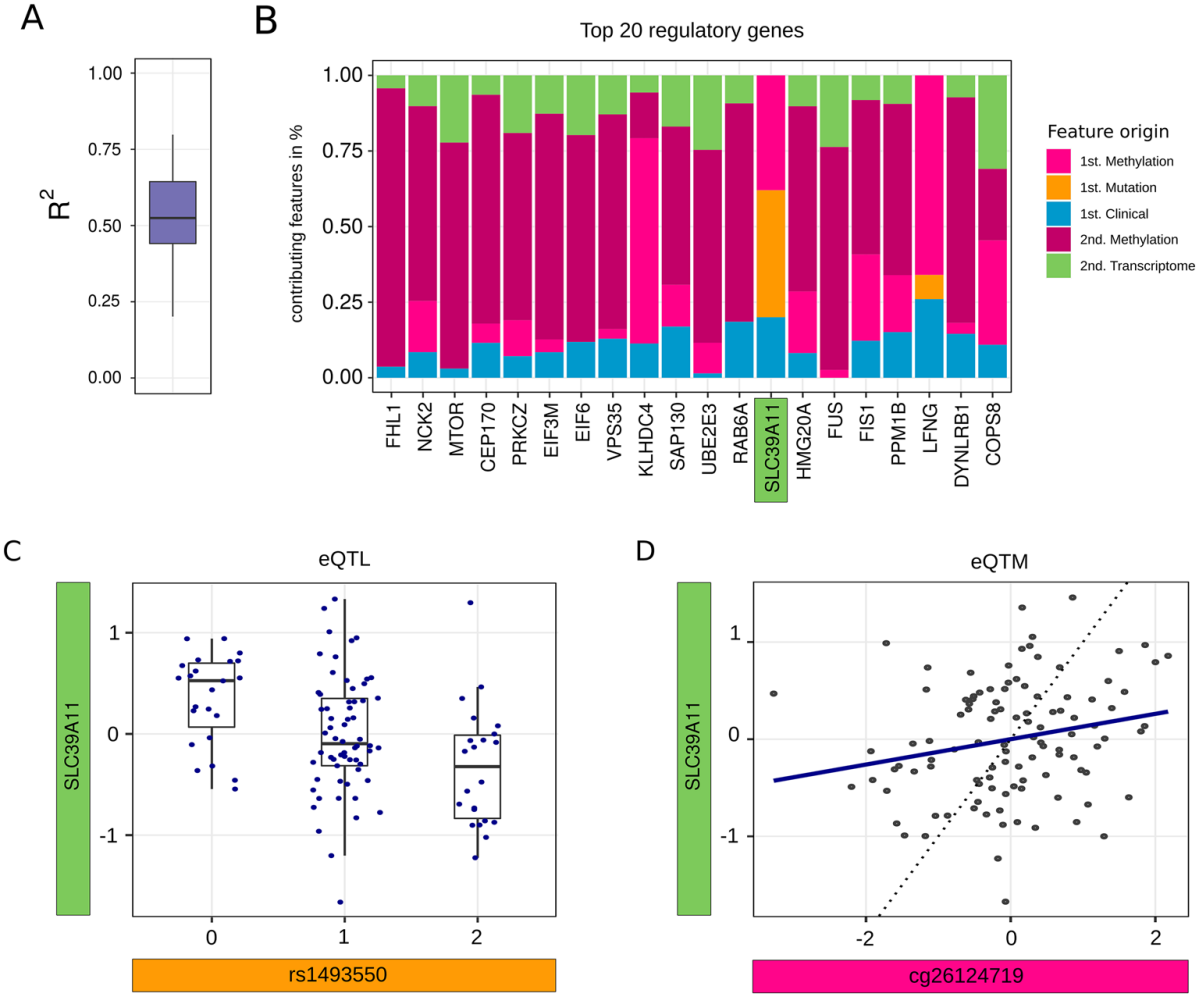
(**A**) Performance of n = 20 genes with the highest betweenness and (**B**) its composition of retained features deriving from omic levels for each gene. Gene expression with possible influence by (**C**) SNP and (**D**) methylation site found with KiMONo; the dotted line represents a correlation of 1.

The top 20 genes identified with the highest betweenness measure were found to exhibit higher performances compared to the average model. ${R}^{2}$ ranged from 0.202 to 0.798 with a median of over 0.525, while the average across all models was 0.539 (Fig. 6A). Further, features selected by the penalty model represented information from many different omic-information levels, across methylation, SNP, gene expression as well as biological clinical information. Methylation sites possessing long-distance effects, gene expression associated over indirect links, and biological data were consistently present for the top 20 hits (Fig. 6B).

The potential of our method becomes apparent when looking at connections found through KiMONo but not pairwise models of MatrixEQTL. After correcting for residual effects of all other features in multilevel models, the connection between the expression of SLC39A11 (Solute Carrier Family 39 Member 11, chromosome 17) and SNP rs1493550 and methylation site cg26124719 located both in an intron became clearly resolved (Fig. 6C,D).

**Table 1:** Top 10 genes of most important nodes within Major Depressive Disorder (MDD) data. Ranking was derived via the nodes betweenness of centrality.

	Gene symbol		Location	MDD	BP	References
1	FHL1	Four and a half LIM domains 1	chrX:136,146,702-136,211,359	No		
2	NCK2	NCK adaptor protein 2	chr2:105,744,912-105,894,274	No		
3	MTOR	Mechanistic target of Rapamycin kinase	chr1:11,106,531-11,262,557	Yes		^32,33^
4	CEP170	Centrosomal protein 170	chr1:243,124,428-243,255,406	No		
5	PRKCZ	Protein kinase C zeta	chr1:2,050,411-2,185,395	No	Yes	^34,35^
6	EIF3M	Eukaryotic translation initiation factor 3 subunit M	chr11:32,583,767-32,606,2	Yes		^36,37^
7	EIF6	Eukaryotic translation initiation factor 6	chr20:35,278,906-35,284,985	No		
8	VPS35	Vacuolar protein sorting-associated protein 35	chr16:46,656,132-46,689,518	Yes		^38^
9	SAP130	Spliceosome-associated protein 130	chr2:127,941,217-128,028,120	Maybe		^39^
10	KLHDC4	Kelch domain containing 4	chr16:87,696,485-87,765,997	Yes		^39,40^

Half of the top 10 hits have been previously linked to depression or pathways involved in the pathogenesis of the disease (see Table 1). Here the top enriched KEGG pathway was endocytosis (FDR = 4.832e−8) which plays a major role in synaptic plasticity, which is an important component in disease development of stress-related disorders, like MDD^27,28^. The second important pathway was autophagy (FDR = 2.606e−6) an essential pathway for the central nervous system and studies have shown the effects of antidepressant treatments on autophagy^29^. Interestingly, among the top 10 pathways was Axon guidance (FDR = 1.054e−3), which has been shown to be a strong risk factor for depression, as stress may affect brain structure and function^30,31^.

## Discussion

We presented KiMONo—a novel prior Knowledge guided Multi-Omics Network inference method. By leveraging prior knowledge, the algorithm builds a statistical model for each gene, selects the most predictive features and uses these to assemble a multi-level network. Within this network nodes represent features of the input omic measurements and links define disease- or context-specific relation between them. Within all the possible linkages of nodes as derived from the prior, our network can be viewed as a subnetwork that is specific to condition-setting, containing only edges between meaningful associations. KiMONo was specifically designed to work on low sample size sets with high-dimensional data originating from a variety of information sources.

We used TCGA data, one of the biggest collections of multi-omic data, as the main evaluation set. For some omic types the data was lacking quality and information depth. For instance, mutation and methylation data were only available in a binarized form. We reasoned that KiMONo enhances the signal by combining various data sources and is therefore well suited for the analysis for this data format. Nevertheless, we also performed our tests on less preprocessed data describing MDD. Even though this data set has a higher dimensionality, we were also able to reproduce the performance behaviour we gained from the TCGA data (see Fig. S5).

In our robustness tests, we showed that, reducing the number of samples barely affected the overall performance of KiMONo on TCGA’s PanCancer subset. When investigating the performance contribution of the mutation features alone, there was even a slight performance increase for low sample sizes. Even though it might be the sole effect of overfitting, we showed that it only occured for sparse binarized data. Hence, removing samples from this sparse matrix directly resulted in setting some features $=0$. Therefore, we not only removed samples but also shrank the feature space. which in turn resulted in less predictive models having slightly better regression performances.

In contrast, we found that the method was more sensitive to noise in the data than to reduced sample size. When increasing the simulated noise, it resulted in a rapid decrease in correctly predicting the gene expression level, as opposed to a moderate decrease when reducing the amount of samples.

Next, we showed that KiMONo was able to find many of the eQTL and eQTM genes (34.5 and 26.7%) that were uncovered by MatrixEQTL using pair and level-wise tests. In addition we found further associations, complementing MatrixEQTL, when deriving regulatory networks in context with all features from all omic levels. It is possible that these features can only be detected when taken into account the context of the underlying omic-crosstalk. Across all top hits in the MDD dataset (Table 1), we observed that relationships from 2nd-order linked genes and methylation sites play an important role. For example, gene SLC39A11 being identified as eQTL and eQTM gene to SNP rs1493550 and methylation site cg26124719. Our results indicate that KiMONO is a powerful method to discover these long-distance and indirect relationships while establishing regulatory networks.

In addition to incorporating second-order links, we also showed the advantage of multivariate models derived from various omic-layers by uncovering relationships that were not found in pairwise models. After correcting for residual effects of every feature except for the one of interest, the connection became clear (Fig. 6C,D). Our approach allows uncovering many more effectors by accounting not only for the covariates but also all other features in a complex multi-omic context.

Applying KiMONo on both TCGA cancer types and MDD, we were able to find previously reported genes that matched well with the underlying disease setting (see Fig. 6B/Table 1). This provided a good evaluation of our method. Among the top hits we also identified genes that have not yet been reported in relation to the studied phenotypes. These genes could be essential for further exploration of the disease mechanisms for better understanding of the underlying molecular interplay.

In summary, we showed KiMONo is a versatile method to derive fully integrated and holistic multi-level networks capturing the data-supported interplay between omics levels. Comprehensive benchmarks demonstrated that KiMONo is more sensitive to noise than to the reduction of samples. Further, application to two human disease settings showed that key nodes of the inferred multi-omics disease networks also play key roles in disease pathophysiologies. Ultimately, the holistic networks inferred using KiMONo may serve as tools to easily uncover key regulatory features, no matter the disease setting or complexity of the data.

## Methods

### The cancer genome atlas data and prior

As a real world example, we applied KiMONo to 12 different datasets of varying complexity. The first 11 datasets were obtained via the PanCancer data from The Cancer Genome Atlas (*TCGA*) data portal^41^. This is one of the most comprehensive multi-omic data sources. This collection contains multi-omic data sets of 4926 samples describing 11 different cancer types—*acute myeloid leukemia (191* samples*)*, *bladder urothelial carcinoma (135* samples*), breast invasive carcinoma (871* samples*), colon adenocarcinoma (421* samples*), glioblastoma multiforme (580* samples*), head & neck squamous cell carcinoma (309* samples*), kidney clear cell carcinoma (496* samples*), lung adenocarcinoma, lung squamous cell carcinoma (344* samples*), ovarian serous cystadenocarcinoma (563* samples*), rectum adenocarcinoma (164* samples*)* and *uterine corpus endometrioid carcinoma (495* samples*).* The portal provides open access to highly preprocessed ‘*level 3′* data of five omic characterizations, *Proteome (*~ *130 proteins)*, *Transcriptome (*~ *16,115 transcripts)*, *Copy Number Variation* (~ *84 CNV*), *Mutation (*~ *39,675 positions)*, *Methylation (*~ *2043 sites)* but also phenotype information in the form of *Clinical* data (*4 variables*). In our analysis we only included samples which were measured across all 5 omic levels, restricting the data sets to 2036 patients across 11 cancer types (see Fig. S1). Beside binarizeing the *Clinical* feature ‘sex’ we also standardized all input features.

In order to assemble the prior knowledge networks for the *PanCancer* cohort, we used both first- and second-order links to connect the *Transcriptome* to all information levels. First-order links to the *Proteome* were generated via the *bioMart* annotation resource. First-order links to *CNV* and *Methylation* were generated via a genomic position-confined prior. Here we used Bioconductor’s R packages *Homo.sapiens*, *GenomicRanges *^42^ and *FDb.InfiniumMethylation.hg19 *43 to* link* copy numbers and methylation sites within a 500 kb range to genes of interest. The *Mutation* data type was already projected to gene identifiers, hence there were no additional preprocessing steps needed. Furthermore, we used experimentally validated associations from *BioGrid*^44^ to create links within the *Transcriptome*. Additionally, we added second-order links to increase the coverage of individual gene models. This was done by connecting genes to first-order linked features of gene neighbours. In a final step, we also connected all features within the *Transcriptome* to the *Clinical* features.

### Major depressive disorder data and prior

In addition to the TCGA data we also used a Major Depressive Disorder (*MDD*) data set as a second real data example. This cohort consisted of 289 caucasian individuals, 160 healthy controls and 129 patients diagnosed with major depressive disorder. Recruitment strategies and further characterization of the *MDD* cohort have been described previously in^26,45^. Three levels of omic information, comprising the transcriptome, methylome and genotype, as well as biological information, were measured for 107 out of 289 individuals, consisting of 33 females and 74 males, distributed over 64 controls and 43 patients. Details on the omic preprocessing can be found in^26,45^.

For generating the prior knowledge first-order links, we annotated gene expression probes and gene symbols using the Re-Annotator pipeline^46^ based on GRCh37 (hg19) RefSeq. Additionally, we annotated methylome, the CpG site probe, and the transcriptomes gene symbol to sequence positions by performing a re-alignment using Bismark^47^. Furthermore, we connected the genes to SNPs and methylation sites within a distance of 10 kbp and 500 kbp, respectively. Second-order links were created between genes via a ‘guilt-by-association’ approach using the BioGrid database. Furthermore, we connected genes with their associated genes methylation site generating, introducing second-order linked methylation sites.

### Performance test

To assess goodness of fit on every gene-level model, we use the r-squared metric measuring how much of the variance of the expression can be explained by the model. We calculate for each model the explained sum of squares $ESS$, defined as $\sum {\left(\widehat{y}-\overline{y}\right)}^{2}$, and total sum of squares $TSS=\sum {\left(y-\overline{y}\right)}^{2}$. Here, $y$ represents the true (measured) and $\widehat{y}$ the predicted gene expression. The amount of variance explained is then given by ${R}^{2}=ESS/TSS$.

In order to approximate each information level contribution to the ${R}^{2}$, we dissect the ${R}^{2}$ and calculate a ${R}_{l}^{2}$ for each *l* omics/clinical level. This is done by calculating the ${R}_{l}^{2}$ via $\widehat{y}\sim {X}^{m}{\beta }^{m}$ and ${y}_{new}=y-{X}^{n}{\beta }^{n}$. Here $m$ defines all features within level $l$ and $n$ denotes all other features, For example in the *PanCancer* data set, to dissect the goodness of fit for contribution of the proteomics level, we corrected the gene expression measurements by the contribution of the other omics layers but not the proteomics level. Then, ${y}_{new}$ and $\widehat{y}$ were further used to estimate a ${R}_{l}^{2}$ which approximates the sole performance of *l*. Finally, ${R}_{l}^{2}=0\iff \sum m=0$, which sets the performance of levels without selected features of a given level *l* to 0.

### Contribution of second order prior links

Overall, we compared two different prior strategies. On the one hand, a prior solely based on the genomic location and annotation databases. Here we annotated protein, methylation, mutation and clinical information to the transcriptome level. On the other hand we generated a prior also including second-order links using the BioGrid^44^ resource. We not only interconnected the transcriptome but also all other layers.We used the *PanCancer breast invasive carcinoma* network models as test scenarios, investigating the impact of the different prior strategies. To evaluate how well each strategy performed, we compared the performance of the models, which explained at least 10% of the variance within the data and the coverage of the inferred networks.

### Robustness to noise and low sample sizes

To benchmark the performance on small data, we simulate data sets with shrinking sample size. Therefore we used the *TCGA breast invasive carcinoma* data and randomly reduced the amount of samples. We repeated each simulation 20 times (except for the case where 100% of the data was available). The final test cases included 10%, 30%, 50%, 70% and 100% of the data. Note, for each generated dataset, KiMONo excludes features with $\sigma =0$.

We followed a similar strategy for benchmarking the robustness of the method with respect to noise. Here we simulated test sets by decreasing the signal to noise ratio. All simulated sets were generated using a subset of the *PanCancer breast invasive carcinoma* data. Random noise was generated using Gaussian noise, $N\left(0,{\sigma }^{2}\right)$ with increasing ${\sigma }^{2}$. Here we simulated noise with $\sigma \in \{\mathrm{0,0.2,0.4,0.6,0.8,1}\}$ and summed noise and original measurements to simulate an increase in noise. For both, we used the above described ${R}^{2}$ and ${R}_{l}^{2}$ metric to evaluate the models' performances, excluding all models ${R}^{2}<0.1$.

### Quantitative trait analyses

In the MDD dataset, we implicitly computed multivariate expression quantitative trait loci in the KiMONo approach, as we impose a genomic proximity prior to link variants and gene expression measurements. Thus, we compared the quantitative trait analysis results of KiMONo to the state-of-the-art pairwise analysis tool, matrixEQTL. Here we used both methods to detect expression quantitative trait loci (eQTL) and expression quantitative trait methylation sites (eQTM) genes within the MDD data set. For the matrixEQTL calculation, we focused on cis-eQTL and cis-eQTM windows of 10 kbp and 500 kbp distance, respectively. Further, we corrected the expressed genes for the covariates, BMI, age, sex and status of the diagnosis, with significance threshold set to $DR<0.05$. In the case of KiMONo, eQTL and eQTM genes are identified via the inferred cross-layer interactions between genes and methylation sites and SNP’s. Here, robustly inferred results were defined as models with ${R}^{2}\ge 0.1$ and the respective cross-layer association of $\ge 0.2$.

### Network analysis

We treated all links between the multiple levels after KiMONo inference as undirected edges, generalizing the multi-layer directed network to a simple single-layer association network representation. To show that the generalized network structure is, like most biological networks, scale-free, we tested goodness of fit to evaluate if the node degree follows a gamma distribution^48^. Furthermore, we used the *betweenness centrality* to estimate the importance of nodes within the single-layer network. The *betweenness centrality* is defined as $\left(v\right)=\sum \frac{{\sigma }_{st}\left(v\right)}{{\sigma }_{st}}$. Here ${\sigma }_{st}\left(v\right)$ defines the shortest path between node s to node t, passing node v.

### Data access

The *PanCancer* data is publicly available via the *TCGA* data portal (downloaded May, 2017). A list of the sampleIDs and cancer types which contained all 5 omic levels can be found in Data S1. The transcriptomic and epigenomic information layer of the MDD cohort can be found at GEO GSE64930 and GSE74414, while the SNP data cannot be provided due to patient privacy regulations.

### Software and prio data sources

KiMONo is freely available as an R package on https://github.com/cellmapslab/KiMONo. We used the Bioconductor’s R packages *Homo.sapiens*, *GenomicRanges* and *FDb.InfiniumMethylation.hg19* to generate the annotation between various omic types within TCGA’s PanCancer data. Furthermore we used the Re-Annotator pipeline^46^ based on GRCh37 (hg19) RefSeq and Bismark^47^ to annotate the MDD data. For state-of-the art eQTL analysis we applied the open source tool matrixEQTL (version 2.3). Pathway annotations were performed via the open source tool pathwaX^13^.

### Ethics approval and consent to participate

No ethics approval was required for the study.

## Supplementary Information

Supplementary Information
