# Supplementary material for: Versatile knowledge guided network inference method for prioritizing key regulatory factors in multi-omics data
Source: Sci Rep. 2021 Mar 24;11:6806. doi: 10.1038/s41598-021-85544-4 (PMC7990936; doi:10.1038/s41598-021-85544-4)
Supplement: Supplementary file 1 — Supplementary Information [file 41598_2021_85544_MOESM1_ESM.pdf]

Supplemental Information

Versatile knowledge guided network inference method for prioritizing key regulatory factors in multi-omics data

Christoph Ogris[1], Yue Hu[1], Janine Arloth[1,2] and Nikola S. Müller[1]

[1]Institute of Computational Biology, Helmholtz Center Munich, Ingolstädter Landstr. 1 85764 Neuherberg, Germany
[2]Department of Translational Psychiatry, Max Planck Institute of Psychiatry, 80804 Munich, Germany

Corresponding authors: Christoph Ogris, Nikola Müller
Institution: Institute of Computational Biology
Address: Helmholtz Center Munich, Ingolstädter Landstr. 1 85764 Neuherberg, Germany
Mail: christoph.ogris@helmholtz-muenchen.de, nikola.mueller@helmholtz-muenchen.de

Supplemental Tables

Gene symbol	#Publication hits	Gene symbol	#Publication hits
BRCA1	271 000	HNRNPA1	5 370
CDK2	74 300	HSP90AA1	4 410
DLG4	1 540	MYC	1 030 000
EP300	11 100	SKP1	14 600
FBXW11	844	UBC	45 600
GRB2	47 300	UBE2I	1 630
HDAC1	41 600	AKT1	52 400

Sup. Table 1: KiMONo identified 14 genes which are identified as important across all 11 panCancer networks. The publication hits define the approximate amount of publication including the gene. These hits were derived using google scholar searches.

Supplemental Figures

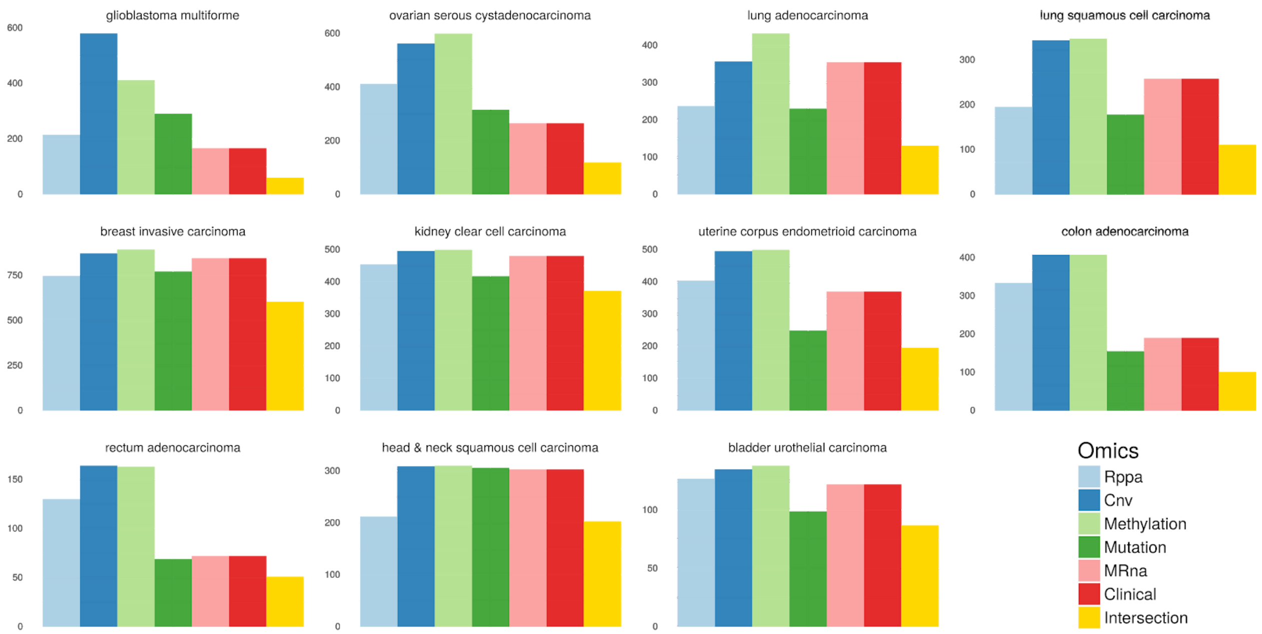

Sup. Figure 1: *Overview of pan cancer data for 11 different cancer types and 6 different data types. Proteomics / Rppa (blue), Copy number variations / CNV (dark blue), Methylation (limegreen), Mutations (dark green), mRNA (pink) and Clinical information (red). The intersection (yellow) denotes the amount of matched samples. In our analysis we only used the samples which were analysed across all levels.*

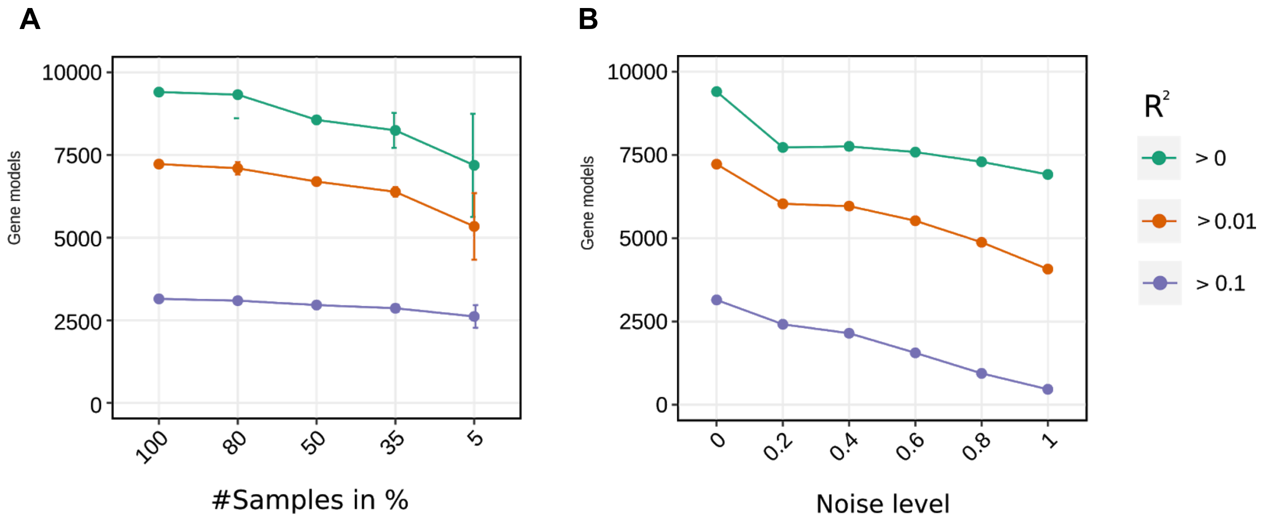

Sup. Figure 2: *Overview of network coverage for sample reduction (A) and noise level (B) benchmarks.*

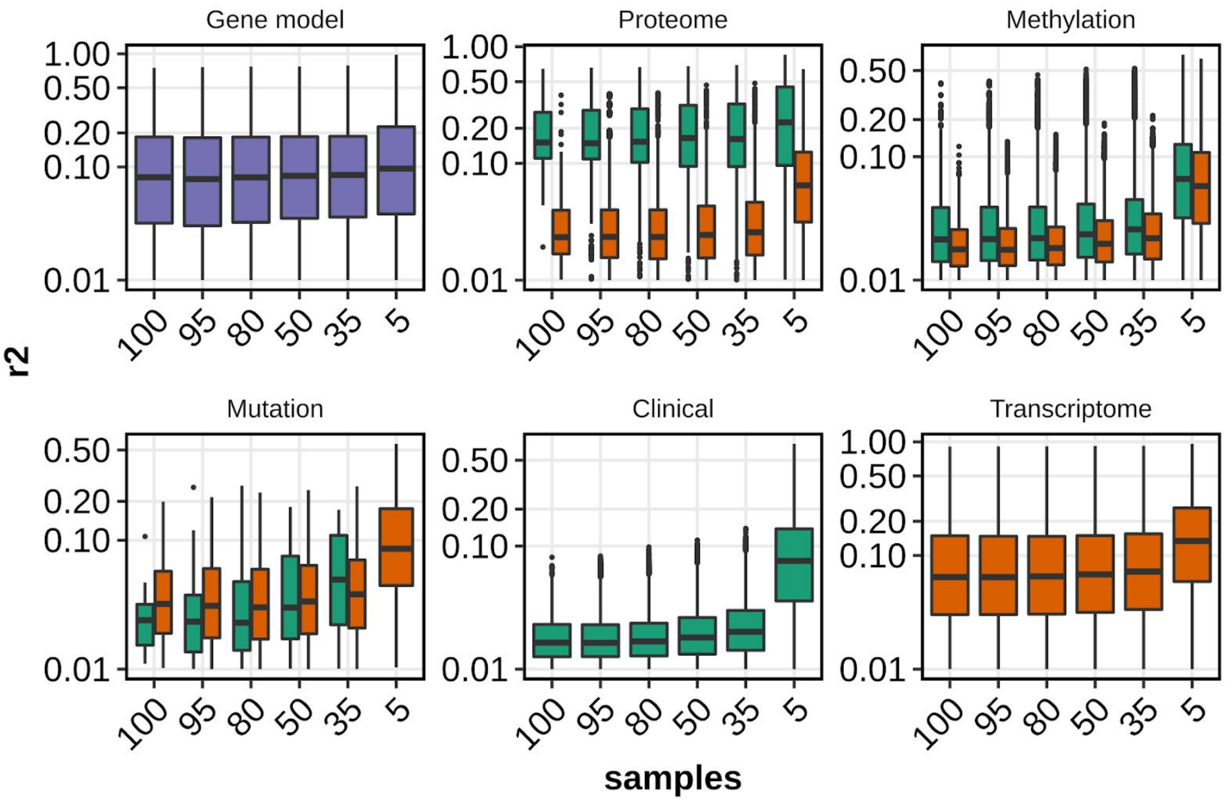

Sup. Figure 3: *Results of benchmarking small sample sizes and different noise levels. Here, we used all inferred models which explain at least 1% of the variance in the data.*

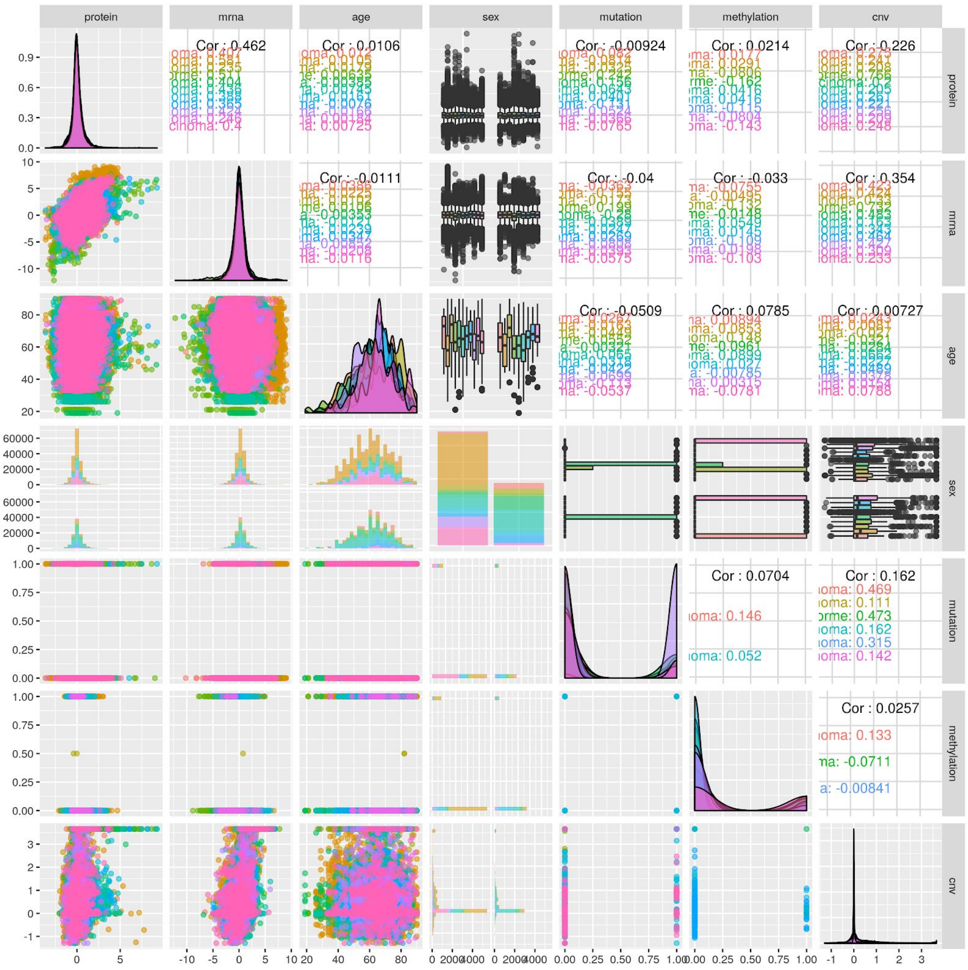

Sup. Figure 4: *Overview of the available TCGA raw omic data for all 10 cancer types. Rows and columns denote the omic levels. For the clinical data level each feature is visualized separately since it consisted of binarized and continuous data.*

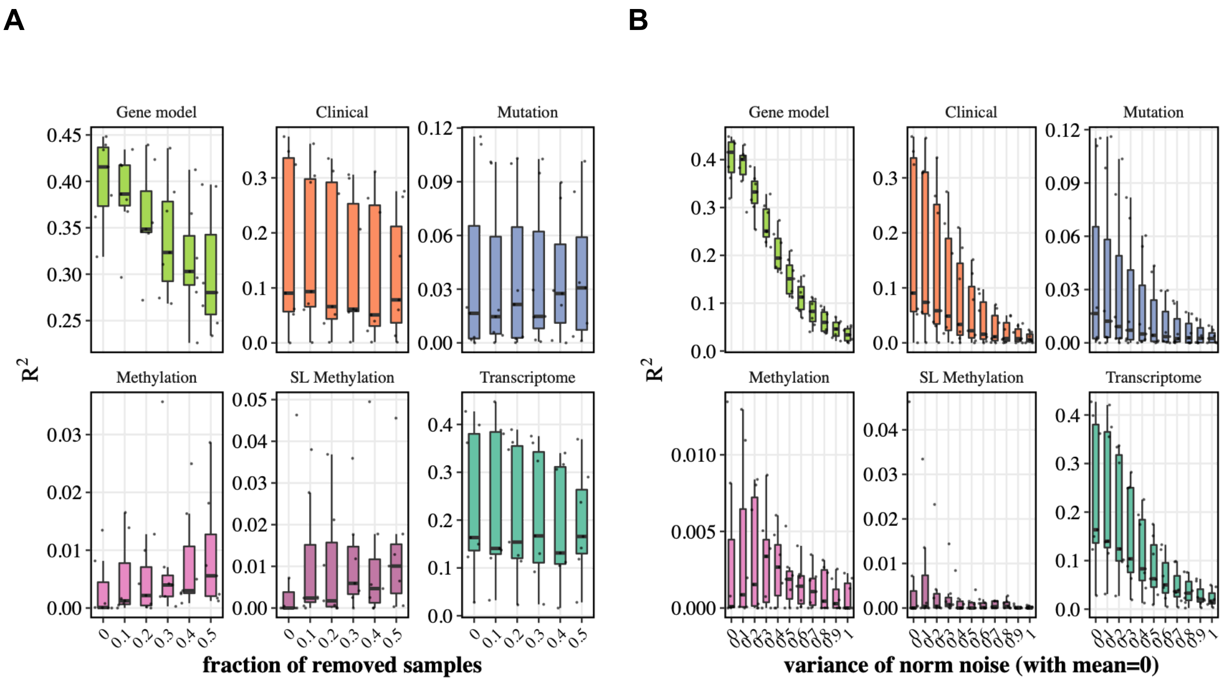

Sup Figure 5: *Robustness benchmark for A) different sample sizes and B) noise levels on MDD data. The boxplots show the performance R^2 of inferred gene models. Panels describing the performance of stand-alone first-order links are displayed first (Clinical, SNPs and Methylation), followed by second-order links (Prior Methylation and Transcriptome). The last panel shows the performance of inferred gene models using all available information layers. A) Data sets with different sample sizes were generated using 10% - 50% of the 107 MDD samples. B) Different test data sets were simulated by adding Gaussian noise with increasing variance. Here, the noise level reflects the σ for ten intensities.*

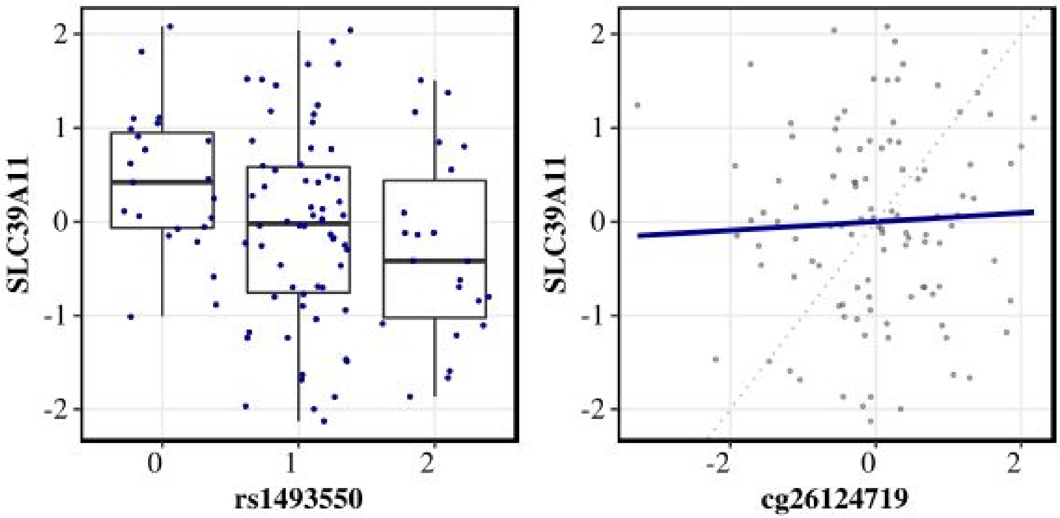

Supp Figure 6: Gene expression with possible influence by C) SNP and D) methylation site found with KiMONo but not with MatrixEQTL before correcting for residual effects - raw data; the dotted line represents a correlation of 1.
